# As questões de uma história global das técnicas

**DOI:** 10.1590/S0104-59702025000100056

**Published:** 2026-02-09

**Authors:** Rafael Dalyson Souza

**Affiliations:** i Programa de Pós-graduação em História das Ciências/Casa de Oswaldo Cruz/Fiocruz. Rio de Janeiro – RJ – Brasil fiocruzrafael@gmail.com

*Global history of techniques* é uma coletânea de destaque no campo da história das ciências lançada em 2024 pela belga Brepols Publishers e organizada por Guillaume Carnino, Liliane Hilaire-Pérez e Jérôme Lamy. Os organizadores são historiadores franceses especialistas no campo da história das ciências e das técnicas, com contribuições conjuntas anteriores, especialmente Hilaire-Pérez e Carnino, em *Histoire des techniques: mondes, sociétés, cultures XVIe-XVIIIe siècle* (Carnino, Hilaire-Pérez, Kobiljski, 2016).

Em seus 45 capítulos, o livro atual reúne um amplo grupo de pesquisadores dedicados a explorar a especificidade da história das técnicas no contexto da ascensão da história global, refletindo, portanto, a característica já presente nas obras anteriores de conjugar diversas regiões geográficas e temporalidades diferentes em torno da temática da história das técnicas. *Global history of techniques*, no entanto, vai além ao reivindicar a especificidade da história das técnicas em relação à história das ciências.

Nesse sentido, os autores discutem, em primeiro lugar, a existência de categorias centrais para a afirmação do campo, como os termos “técnica” e “tecnologia”. Este último, até o século XVIII, referia-se a um discurso sobre a técnica, ou seja, uma ciência voltada para as práticas, artes e manufaturas. Com a ascensão da indústria no século XX, entretanto, essa acepção original caiu em desuso, e o termo passou a designar uma ciência aplicada. Em contrapartida, os autores propõem o uso da categoria “técnica”, entendida como atividades humanas que envolvem ou não o uso de objetos, funcionando como mediação entre o mundo e os artefatos (Carnino, Hilaire-Pérez, Lamy, 2024, p.12). Essa abordagem dialoga com alguns pioneiros do campo, aliás referenciados na obra, como Lucien Febvre (1935), que entendia técnica tanto como a profissão, a exemplo da profissão de operário, quanto como os objetos de que faz uso. Outro exemplo é a contribuição de Marcel Mauss (2003), cuja edição original é de 1950, que ampliou o conceito de técnica para abranger também os movimentos do corpo, como nadar, por exemplo.

Outra importante discussão presente no livro relacionada à afirmação da história das técnicas está na crítica direcionada à perspectiva difusionista no campo da história das ciências, que assume uma circulação fácil e homogênea das ciências e das técnicas. Os organizadores questionam essa visão, enfatizando que as técnicas não apenas circulam, mas também são apropriadas, transformadas e, frequentemente, ignoradas, contribuindo também para a produção de desigualdades (Carnino, Hilaire-Pérez, Lamy, 2024, p.17).

Os capítulos exploram a diversidade de contextos geográficos e temporais, alinhando-se ao objetivo de compreender formas variadas de apropriação técnica, buscando ir além da ideia de difusão. A obra está organizada em três partes: “Techniques across the world” (Parte 1), “Artefacts, processes, sectors” (Parte 2) e “Connections” (Parte 3). A estrutura busca evitar divisões geográficas tradicionais, como Ocidente e Oriente, priorizando temáticas específicas.

A primeira parte, por exemplo, aborda técnicas desde a Oceania, passando por América Latina, Oriente Médio, Magreb, Ásia, Europa e EUA. Organizada de modo não evolutivo, “Techniques across the world” apresenta questões importantes como o encontro de culturas por meio da técnica, a discussão historiográfica sobre o tema na América Latina e o desenvolvimento da noção americana de tecnociência. “Artefacts, processes, sectors” examina artefatos e setores específicos, como a indústria têxtil, técnicas de pesca, telecomunicações e engenharia. A última parte aborda temas contemporâneos, incluindo técnicas em contextos de guerra, feminismo, reciclagem e obsolescência programada. Os capítulos buscam, no geral, atender ao chamado da história global para compreender escalas mais abrangentes, como, por exemplo, as diferentes cronologias tecnológicas e as diversas formas de reciclagem e de reparo de objetos.

Entre os capítulos destaca-se “Latin America in the global history of technology”, do historiador David Pretel, que propõe uma perspectiva regional para a história das técnicas na América Latina. Nele, Pretel (2024), professor da Universidad Autónoma de Madrid em História Econômica, revisita debates como a teoria da dependência, bastante conhecida nos anos 1960, e a posterior crítica desenvolvida a essa teoria pela historiografia das técnicas fundamentada no nacionalismo, isto é, na afirmação de uma técnica nacional. O autor argumenta que a ênfase na afirmação de um desenvolvimento tecnológico nacional obscureceu as relações entre o global e o local, e defende que o estudo das histórias localizadas, regionais, atento às assimetrias, é essencial para compreender o papel da América Latina no cenário global (p.246).

Em síntese, a obra mais recente dos historiadores Guillaume Carnino, Liliane Hilaire-Pérez e Jérôme Lamy oferece contribuição significativa ao campo, propondo análise que transcende a simples circulação das técnicas para investigar suas apropriações, transformações e implicações nas dinâmicas globais. É leitura indispensável para jovens pesquisadores interessados em história das ciências e das técnicas, ampliando as bases para debates historiográficos sobre a relação entre o local e o global.

## References

[B1] CARNINO Guillaume, HILAIRE-PÉREZ Liliane, KOBILJSKI Aleksandra (2016). Histoire des techniques: mondes, sociétés, cultures XVIe-XVIIIe siècle.

[B2] CARNINO Guillaume, HILAIRE-PÉREZ Liliane, LAMY Jérôme (2024). Global history of techniques.

[B3] FEBVRE Lucien (1935). Réflexions sur l'histoire des techniques. Annales d'histoire économique et sociale.

[B4] MAUSS Marcel (2003). Sociologia e antropologia.

[B5] PRETEL David, Carnino Guillaume, Hilaire-Pérez Liliane, Lamy Jérôme (2024). Global history of techniques.

